# Supplementary material for: Combined Inhibition of Hedgehog and HDAC6: In Vitro and In Vivo Studies Reveal a New Role for Lysosomal Stress in Reducing Glioblastoma Cell Viability
Source: Int J Mol Sci. 2023 Mar 17;24(6):5771. doi: 10.3390/ijms24065771 (PMC10051748; doi:10.3390/ijms24065771)
Supplement: Supplementary file 1 [file ijms-24-05771-s001.zip › ijms-2218900-supplementary.pdf]

Table S1. List of antibodies and their relative dilution. Wester-blot (WB), immunohistochemistry (IHC) and immunofluorescence (IF) analyses.

Antibody	Brand	Protocol	Dilution	Model
Rabbit anti LC3A/LC3B	Immunological Sciences (Roma, Italy)	WB<br>IHC	1:500<br>1:60	U87-MG
Rabbit anti LAMP1	Immunological Sciences (Roma, Italy)	WB<br>IHC	1:100<br>1:500	U87-MG
Rabbit anti GAPDH	Santa Cruz Biotechnology (Santa Cruz, CA, USA)	WB	1:1000	U87-MG
Mouse anti p-AKT	Cell Signaling (Danvers, MA, USA)	WB	1:1000	U87-MG
Rabbit anti AKT	Cell Signaling (Danvers, MA, USA)	WB	1:1000	U87-MG
Mouse anti p-ERK 1/2	Cell Signaling (Danvers, MA, USA)	WB	1:1000	U87-MG
Rabbit anti ERK 1/2	Cell Signaling (Danvers, MA, USA)	WB	1:1000	U87-MG
Goat anti-rabbit IgG-HRP	Immunological Sciences (Roma, Italy)	WB	1:200	U87-MG
Goat anti-mouse IgG-HRP	Immunological Sciences (Roma, Italy)	WB	1:5000	U87-MG
IgG anti-rabbit HiLyte Flour 488	Thermo Fisher Scientific Inc. (Waltham, MA, USA)	IF	1:500	U87-MG
IgG anti-mouse Alexa Fluor 546	Thermo Fisher Scientific Inc. (Waltham, MA, USA)	IF	1:7000	U87-MG
Mouse anti-GFP	Sigma-Aldrich (Saint Louis, MO)	IF	1:1000	Zebrafish
Rabbit anti-SQSTM1/p62	Immunological Sciences (Roma, Italy)	IF<br>WB	1:200<br>1:1000	Zebrafish U87-MG
Alexa 488-conjugated goat anti-mouse IgG	Invitrogen Life Technologies (Carlsbad, CA, USA)	IF	1:400	Zebrafish
Alexa 546-conjugated goat anti-rabbit IgG	Invitrogen Life Technologies (Carlsbad, CA, USA)	IF	1:400	Zebrafish

A B

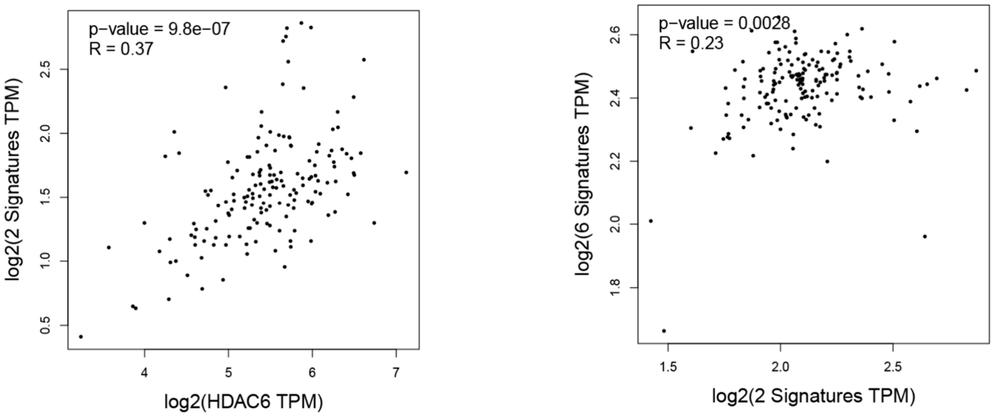

Supplementary Figure S1. ***Hh, HDAC6*** **and sphingolipids enzyme correlation in GBM datasets and U87-MG.** A) GEPIA-2 correlation analyses between the expression levels of *HDAC6* (HDAC6 TPM) and *GLI1* and *PTCH1* (2 signature TPM). B) orrelation analyses between the expression levels of *HDAC6* and *GLI1* (2 signature TPM) and expression of the genes of sphingolipid metabolism (6 signature *TPM; CERS1, SGMS1;SPHK1;SPTLC1;UGCG;SGPP1*). GBM: Glioblastoma; TPM: transcript per millions. Correlation analyses: Spearman correlation. ***p<0.001.

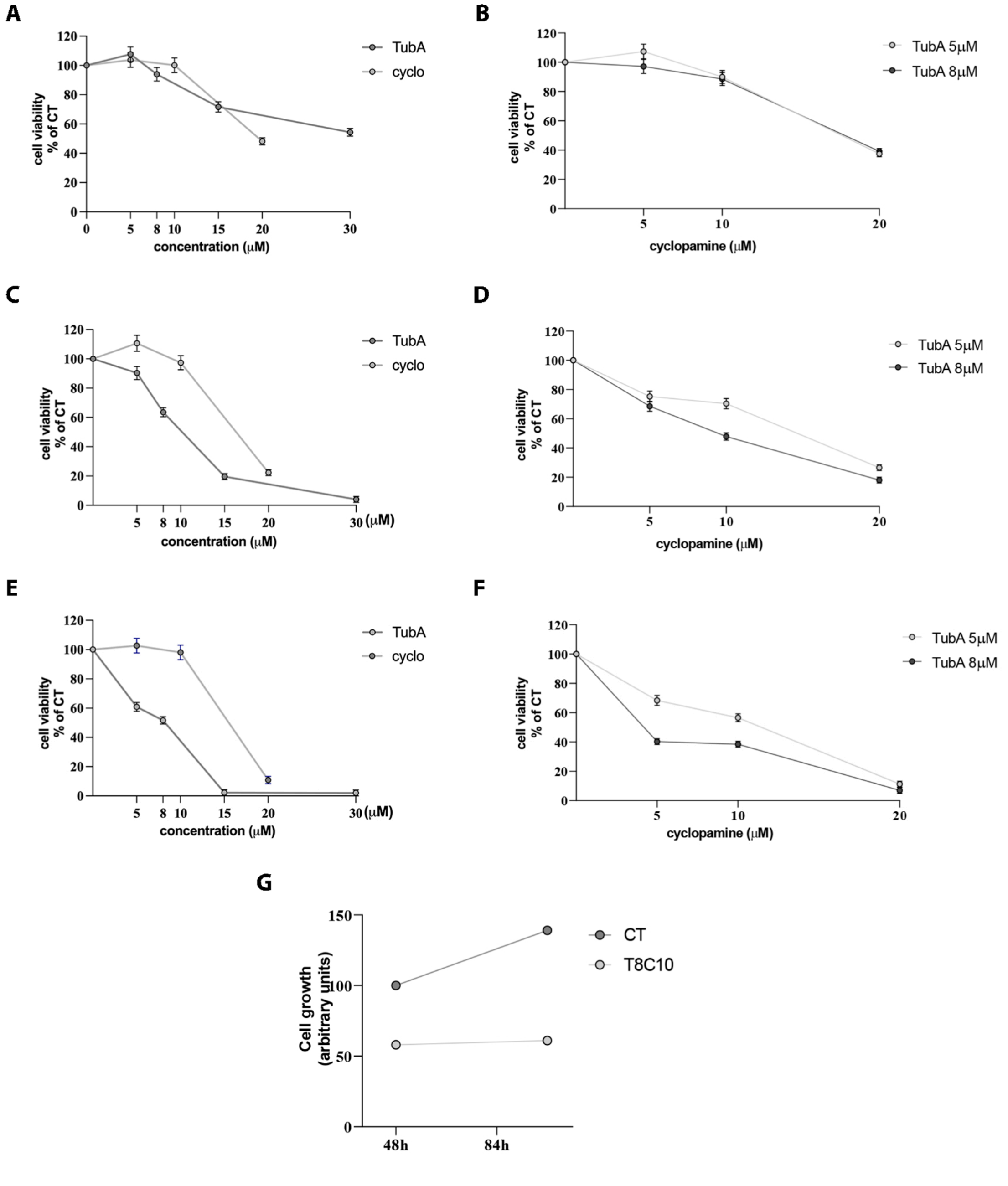

Supplementary Figure S2. Setting conditions for TubA and cyclo administration in U87-MG cells: Effects of TubA and cyclo on U87-MG cells viability alone (**A, C, E**) and in combination (**B, D, F**) after 24 h (**A** and **B**), 48h (**C** and **D**) and 72h (**E** and **F**) of treatment. **G)** Effect of 48h combined treatment with 8 μM TubA and 10 μM cyclo following 24h treatment withdrawal on cell viability. Data are presented as cell growth (arbitrary units, A.U.) ± standard deviation, at each time point. TubA: TubastatinA; cyclo:cyclopamine.

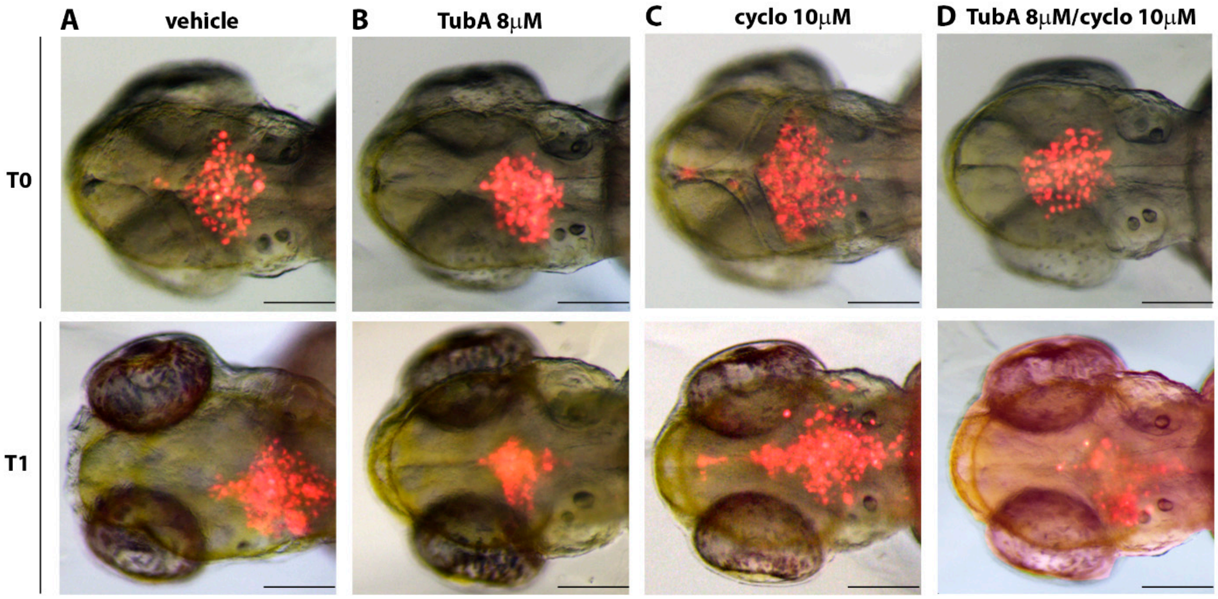

Supplementary Figure S3. Xenotransplantation of U87 GBM cells treated with *Hh* and HDAC6 inhibitiors in zebrafish hindbrain A) Representative images of the head region of zebrafish embryos xenotransplanted with la-belled U87-MG cells pre-treated with B) CT, C) T8, D) C10 and E) T8C10 a T0 or T1 (24 hpi). Scale bare indicates 100 μm.

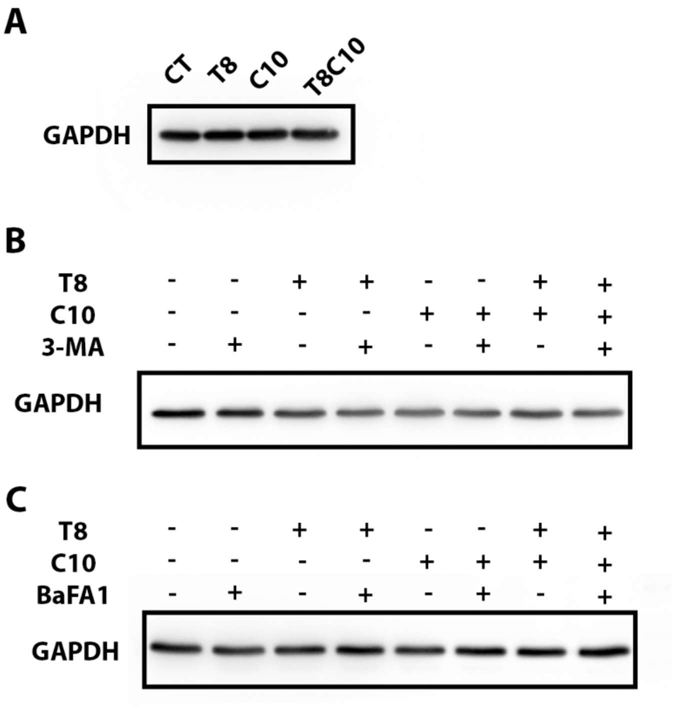

Supplementary Figure S4. Western blot analyses of GAPDH in the U87-MG cell lines. A) GAPDH levels of U87-MG cell line used in the experiment for the analyses of pERK and pAKT (Figure 1G) and GAPDH levels of U87-MG cell line in the experiments with the treatment with **B**) 3-MA (Figure 2A) and **C**) BafA1 (Figure 2B). CT: vehicle; TubA: TubastatinA; cyclo: cyclopamine; 3-MA: 3-methyladenine; BafA1: Bafilomycin A1. + and – indicate the presence or the absence of the chemical in the cell medium respectively.

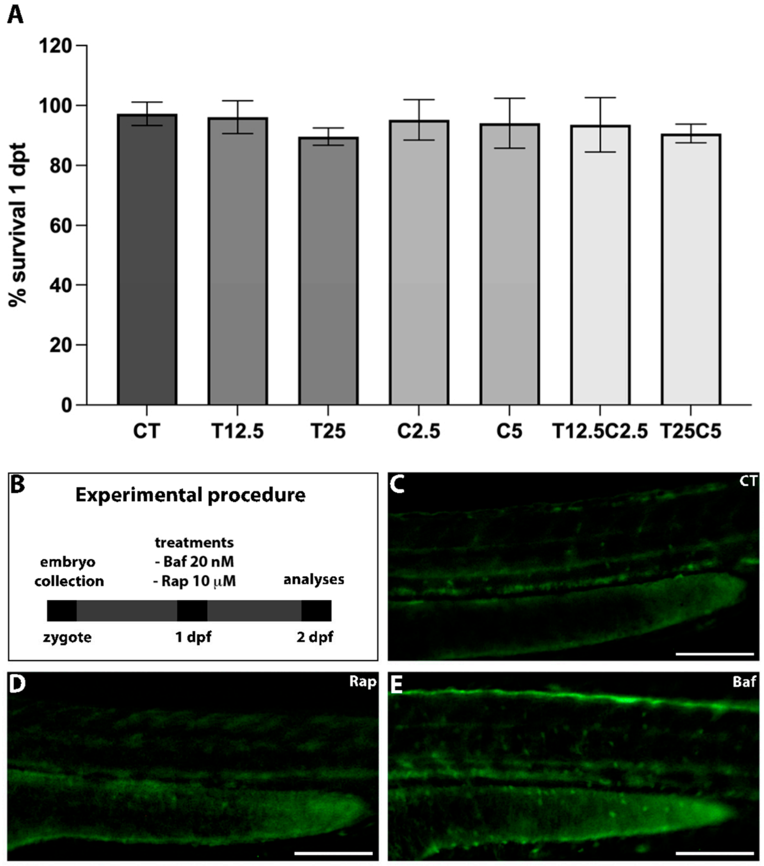

Supplementary Figure S5. Setting doses for pharmacological treatment and effect of autophagy modulators in zebrafish embryos A) Survival analyses of embryos treated with different concentration of TubA and cyclo, alone or in combination setting. **B)** Schematic representation of the pharmacological treatment with autophagy modulators in the Tg(*CMV-lc3b:GFP*) line. **C-E**) Representative images of the trunk region of embryos treated with **B**) CT; **C**) 1 μM Rap; **D**) 20 nM BafA1. Scale bar indicates 100 μm. CT: control; T: TubastatinA; C: cyclopamine; Rap: rapamycin; BafA1: Bafilomycin A1.
